# Effect of exercise and antioxidant supplementation on cellular lipid peroxidation in elderly individuals: Systematic review and network meta-analysis

**DOI:** 10.3389/fphys.2023.1113270

**Published:** 2023-02-14

**Authors:** Chunxia Ni, Yiyi Ji, Keke Hu, Kai Xing, Yining Xu, Yanan Gao

**Affiliations:** ^1^ Faculty of Sports Science, Ningbo University, Ningbo, China; ^2^ College of Music, Ningbo University, Ningbo, China

**Keywords:** exercise, lipid peroxidation, network meta-analysis, elder, systematic reveiw

## Abstract

**Background:** The viewpoints of previous studies on the correlation between exercise and cellular lipid peroxidation are contradictory from many perspectives and lack evidence for elder individuals. A new systematic review with network meta-analysis is necessary and will have significant practical value to provide high-quality evidence in the development of exercise protocols and an evidence-based guide for antioxidant supplementation for the elderly.

**Aims:** To identify the cellular lipid peroxidation induced by different types of exercise, with or without antioxidant supplementation, in elderly individuals.

**Methods:** Randomized controlled trials that recruited elderly participants and reported cellular lipid peroxidation indicators and were published in peer-reviewed journals in English were searched by a Boolean logic search strategy and screened in the databases PubMed, Medline, Embase, and Web of Science. The outcome measures were the biomarkers of oxidative stress in cell lipids in urine and blood, namely F2-isoprostanes, hydrogen peroxide (LOOH, PEROX, or LIPOX), malondialdehyde (MDA), and thiobarbituric acid reactive substances (TBARS).

**Result:** 7 trials were included. A combination program of aerobic exercise (AE), low-intensity resistance training (LIRT), and a placebo intake (Placebo) and a combination program of aerobic exercise, low-intensity resistance training, and antioxidant supplementation (S) had the most and sub-most potential to dampen cellular lipid peroxidation (AE + LIRT + Placebo: 0.31 in Rank 1 and 0.2 in Rank 2; AE + LIRT + S: 0.19 in Rank 1 and 0.20 in Rank 2); A placebo intake (Placebo) and a blank intervention without exercise (NE) had the most and sub-most potential to induce an enhancement of cellular lipid peroxidation (Placebo: 0.51 in Rank 9 and 0.16 in Rank 8; NE: 0.16 in Rank 9 and 0.28 in Rank 8). All included studies had an unclear risk of selecting reporting. There were no high confidence ratings in all the direct and indirect comparisons, 4 comparisons in the direct evidence structure and 7 comparisons in the indirect evidence structure had moderate confidence.

**Conclusion:** A combined protocol consisting of aerobic exercise and low-intensity resistance training is recommended to dampen cellular lipid peroxidation. Extra antioxidant supplementation might be unnecessary if an elderly individual has enough aerobic and resistance exercise.

**Systematic Review Registration:** CRD42022367430

## 1 Introduction

Oxidative stress continuously generates hydrogen peroxide (H_2_O_2_), superoxide, hydroxyl free radicals, and singlet oxygen as one of the normal metabolic processes. These chemical reactive molecules containing oxygen are members of free radicals and non-radical derivatives of oxygen, and they could be called reactive oxygen species (ROS) (Bergamini et al., 2004). Biologically, ROS are highly reactive chemicals with complicated production pathways that could induce cellular lipid peroxidation (LIPOX) (Hayyan et al., 2016). The oxidation of the cell membrane induced by ROS in the process of LIPOX is the process in which the ROS reacts with macromolecular substances such as phospholipids, enzymes, and cell membrane receptors to form LIPOX by-products. The lipid peroxidation products produced by this process mainly include malondialdehyde (MDA) and 4-hydroxynonenal (HNE), which will change the fluidity and permeability of the cell membrane, destroying the lipid bilayers of the cell membrane, and then change the cell structure and function or even cell death (Halliwell and Chirico, 1993). In recent decades, the impact of physical exercise on oxidative stress has attracted extensive attention, although the understanding of exercise and oxidative stress has advanced, questions remain about whether exercise-induced increases in ROS production are beneficial to health or not (Powers et al., 2020). Several previous studies have given some methodological advice, as well as several pieces of evidence with high-quality, suggesting that whether the effect of exercise-induced oxidative stress is positive or negative depends on the health status of individuals and calling for an integrative approach of multi-markers to measure the oxidative stress in response to physical exercise instead of selecting only one biomarker to quantify physical exercise-induced oxidative stress in humans (Lu et al., 2021; Squillacioti et al., 2021).

Previous studies have also identified that during the oxygen refusion for anoxic tissues, the cellular lipid peroxidation, especially on biomembranes, induces a series of negative changes in cellular functions, which contain increased permeability of cellular membranes, a decreased transport rate of calcium of the sarcoplasmic reticulum, changes in the function of mitochondrial, and toxic metabolic by-products produced through other metabolic pathways (Girotti, 1985; Ronguist, 1992). Hypoxia of body tissue usually occurs during exercise due to blood redistribution, and lipid peroxidation might occur when oxygen is reinfusing into the body’s anoxic tissues (Halliwell and Chirico, 1993). Previous studies have demonstrated that physical activity may be one of the key factors that change cell function induced by exercise (Fogarty et al., 2011). These changes include the swelling of cell mitochondria, the decrease in cell membrane fluidity, and finally lead to edema, proteinuria, hemolysis, and even rhabdomyolysis (Holt et al., 1999; Qi et al., 1999; Solin et al., 2001; Vincent et al., 2002).

At present, the viewpoints of studies on the correlation between exercise and cellular lipid peroxidation are contradictory from many perspectives. Some previous studies reported an enhancement in cellular lipid peroxidation induced by exercise. For example, some research groups used ethane and pentane in the gas of expiration as indicators of lipid peroxidation, respectively, and found that exercise could result in an enhancement of lipid peroxidation in body cells (Gee and Tappel, 1981; Davies et al., 1982; Dillard and Tappel, 1989). A study published in 1985 by Rhee’s team found that electrical stimulation could induce a decrease in the activity of catalase (CAT) in skeletal muscle (Rhee et al., 1985). And a study by Teixeira’s team claimed that despite the enhanced levels of antioxidants, individuals undergoing regular strenuous exercise exhibited more oxidative stress than sedentary individuals (Teixeira et al., 2009). Moreover, malondialdehyde (MDA), an important indicator of lipid peroxidation, has also been reported a significant increase after a maximum effort of exercise under an intensity of VO_2max_ (Lovlin et al., 1987) and a significant positive correlation with the blood lactic acid, which increases continually during high-intensity interval exercise (Faruk Ugras, 2013). The potential mechanism of the cellular lipid peroxidation enhancement might come from the following perspectives. First, during intense exercise, the oxygen consumption of the body increases quickly and the capacity of the antioxidant enzyme system is limited, making the body unable to regulate and control cellular lipid peroxidation (Alessio and Goldfarb, 1988; Leaf et al., 1997). For example, the antioxidant enzymes in skeletal muscle cells include superoxide dismutase (SOD, Mn-SOD in mitochondria, Cu-SOD, and Zn-SOD in cytoplasm), whose main function is to convert the more active ROS to the less active ROS (H_2_O_2_), catalase (CAT), whose main function is converting H_2_O_2_ to H_2_O, and glutathione peroxidase (GPx), whose main function is to help CAT convert H_2_O_2_ to H_2_O (Quindry et al., 2013). SOD and GPx are hydrophilic, whereas CAT is lipoedes. Second, during strenuous exercise, the depletion of the body’s energy substrate and the disturbance of the intracellular oxidative mechanism, such as the change in the ratio of the free radical scavenging enzyme such as nicotinamide adenine dinucleotide (NADH) and nicotinamide adenine dinucleotide phosphate (NADPH), lead to the enhancement of lipid peroxidation (Krotkiewski and Brzezinska, 1996; Olek et al., 2004). Last, some enzymes in serum1, such as creatine kinase (CK), creatine kinase isoenzymes (CKMB), lactate dehydrogenase (LDH) and other isoenzymes, increase acutely after a single bout of exercise, leading to an enhancement of lipid peroxidation in the resting state after exercise, which could also be called “exercise-induced lipid peroxidation” (Kanter et al., 1988; Aslan et al., 1998; Ghiasi et al., 2015).

Besides, some other previous studies have demonstrated that cellular lipid peroxidation would be dampened after exercise, especially long-term endurance exercise, the definition of which is an exercise with aerobic metabolism as the main energy source such as jogging, cycling, or swimming, more than 150 min per week, and last at least 12 weeks (Alessio and Goldfarb, 1988; Toskulkao and Glinsukon, 1996; Aksoy et al., 2006). For example, Fukai’s team identified that there was no significant difference in liver lipid peroxidation indicators between the rats that conducted an endurance training protocol and those that conducted a control intervention with no exercise (Fukai et al., 2005). The potential mechanism of cellular lipid peroxidation dampening induced by long-term endurance exercise could also come from different perspectives. On one hand, long-term endurance exercise increases the number or volume of mitochondria in body cells, giving these cells a stronger aerobic metabolic capacity, which could lead to the weakening of cellular lipid peroxidation or the enhancement of anti-lipid peroxidation (Davies et al., 1981; Salminen and Vihko, 1983; Venditti and Di Meo, 1996; Taysi et al., 2008). On the other hand, the activity of catalase (CAT) in skeletal muscle increases after long-term endurance exercise (Miyazaki et al., 2001; Sadegh Ghomi et al., 2022).

After considering the controversy within the present studies, as well as most of the existing evidence accumulated in studies whose subjects are athletes, adults, and animals, the cellular lipid oxidative stress induced by different types of exercise in elderly individuals with or without antioxidant supplementation remains unknown. Moreover, the existing evidence on the effect of antioxidant supplementation on exercising individuals is also contradictory. It seems logical, though, to dampen oxidative stress by taking antioxidant vitamins, such as vitamin C and vitamin E. However, studies have not found that this protocol can effectively reduce oxidative stress biomarkers in the human body (Gleeson et al., 2004; Nieman et al., 2004). Some previous studies found that taking these vitamins in RDA will increase oxidative stress, which is opposite to the effect expected, which is to achieve anti-oxidative stress (Gomez-Cabrera et al., 2008). And some other previous studies have claimed that exercise-induced oxidative stress induced more anti-oxidant mechanisms to trigger the adaptive reaction of the body, which could enhance the endogenous ability of antioxidation. However, the enhancement of endogenous ability for antioxidation will be damped by antioxidation supplementation and then the potential benefit of exercise will be decreased (Ristow et al., 2009).

To sum up, on one hand, for a comprehensive understanding of exercise-induced oxidative stress in the elder population, the information about exercise-induced lipid peroxidation is still insufficient. On the other hand, the effect of exercise on lipid peroxidation in the elderly depends on many factors, which included health status, exercise protocol, and the use of antioxidant supplements. Therefore, it is necessary to conduct a systematic review with meta-analysis to synthesize the existing evidence to determine whether exercise is a friend of the prevention of oxidative stress in elderly individuals and the possible influence of antioxidant supplementation. This systematic review aims to identify the cellular lipid peroxidation, which is induced by different types of exercise that with or without antioxidant supplementation, in elderly individuals. This is the first PROSPERO registered systematic review with network meta-analysis, which has a significant practical value. The results of this review could provide professionals with high-quality evidence to help them develop appropriate exercise protocols for elder individuals and give the elderly an evidence-based guide for the use of antioxidant supplements.

## 2 Methods

### 2.1 Protocol and registration

This systematic review was written based on the reporting systematic reviews incorporating network meta-analyses of healthcare intervention guidelines, which was an extension statement for the Preferred Reporting Items for Systematic Reviews and Meta-Analysis (PRISMA) (Hutton et al., 2015). The eligibility criteria, exclusion criteria, and search strategy were proposed and agreed on by two authors (Chunxia Ni and Yanan Gao). The PROSPERO registration number was CRD42022367430.

### 2.2 Eligibility criteria (PICOS)

#### 2.2.1 Population (P)

This systematic review included studies of trials with participants aged over 60 years old on average and without any musculoskeletal disease or clinical exercise contraindication. Elder individuals with obesity, metabolic syndrome, or chronic diseases such as chronic kidney diseases but not prohibited from exercise, were eligible for this systematic review.

#### 2.2.2 Interventions (I)

This systematic review included studies of trials with interventions of the following basic categories: 1) aerobic exercise under any intensity, with the abbreviation being “AE”; 2) anti-oxidative supplementation, with the abbreviation being “S”; 3) high-intensity resistance training, in which the Rating of Perceived was more than 8 (10 in total) or the relative load was equal or more than 80% 1 RM, with the abbreviation being “HIRT”; 4) low-intensity resistance training, in which the Rating of Perceived was equal or less than 8 (10 in total) or the relative load was less than 80% 1 RM, with the abbreviation being “LIRT”; 5) Placebo intake, with the abbreviation being “Placebo”. If an intervention combined more than one of the categories above, a plus sign was used to connect the abbreviations. For example, the abbreviation “S + AE + LIRT” referred to a combination of anti-oxidative supplementation, aerobic exercise, and low-intensity resistance training.

#### 2.2.3 Comparators (C)

Trials in whose control groups the participants were asked to maintain a current physical activity or to take some activities under very low intensity such as stretching or relaxation were included as the comparators of this systematic review, with the abbreviation being “NE”. All the interventions mentioned above could also be regarded as comparators.

#### 2.2.4 Outcomes (O)

The outcome measures were biomarkers of oxidative stress in cell lipids. The laboratory samples of the outcome measures could be urine or blood. The biomarkers of oxidative stress in cell lipids were F2-isoprostanes, hydrogen peroxide (also called LOOH, PEROX, or LIPOX), malondialdehyde (MDA), and thiobarbituric acid reactive substances (TBARS), which also can reflect the level of MDA in plasma (Ohkawa et al., 1979).

#### 2.2.5 Study (S)

Only studies of randomized controlled trials (RCTs) were eligible for this systematic review, and all the eligible studies must be published in peer-reviewed journals with English as the publication language.

#### 2.2.6 Exclusion criteria

Trials were excluded if: 1) participants had musculoskeletal diseases or were clinically exercise contraindications; 2) the average age of participants below 60; 3) participants were asked to have anti-oxidative substance injections during the process of intervention; 4) just published abstracts or lack of original data; 5) outcome measures were ineligible; 6) not published in English.

### 2.3 Information sources

A comprehensive, reproducible search was performed on the databases of PubMed, Medline, Embase, and Web of Science from January 1990 to October 2022. Reference lists were also searched for grey literature. If data were insufficient, the authors were contacted and the missing data were requested.

### 2.4 Search

A Boolean logic search was conducted based on some principles: 1) have “exercise” or “training” in the title; 2) have “randomized” or “randomised” in the abstract; 3) have “F2-isoprostanes” or “hydrogen peroxide” or “LOOH” or “PEROX” or “LIPOX” or “malondialdehyde” or “malonaldehyde” or “MDA” or “TBARS” in the abstract or title; 4) without “review” or “design” or “protocol” or “meta-analysis” in the title; 5) without “rat(s)”, “mouse”, “mice”, “dog(s)” in the title.

In PubMed, the search term was “((exercise) OR (training) [Title]) AND ((randomized) OR (randomised) [Abstract]) AND ((F2-isoprostanes) OR (hydrogen peroxide) OR (LOOH) OR (PEROX) OR (LIPOX) OR (malondialdehyde) OR (malonaldehyde) OR (MDA) OR (TBARS) [Titile/Abstract]) NOT ((review) OR (design) OR (protocol) OR (meta-analysis) OR (rat) OR (mouse) OR (mice) OR (dog)[Title])”, while in Medline, Embase, and Web of Science, the search term was “(exercise OR training TI) AND (randomized OR randomised AB) AND (F2-isoprostanes OR hydrogen peroxide OR LOOH OR PEROX OR LIPOX OR malondialdehyde OR malonaldehyde OR MDA OR TBARS AB) NOT (review OR design OR protocol OR meta-analysis OR rat OR mouse OR mice OR dog TI)”.

Two independent authors (Chunxia Ni and Keke Hu) screened all the titles of the searched studies before the abstract screening. A third independent librarian (Yining Xu) was invited to check synonyms and terms to increase the sensitivity and specificity.

### 2.5 Study selection

The screening of the eligible studies was conducted in the title, abstract, and full text to guarantee that all the potentially eligible studies could be included in this systematic review.

All searched studies were imported into EndNote 20 (Thomson Reuters, Carlsbad, CA, United States) to further screen and remove duplicates. In the first step, two independent authors (Chunxia Ni and Keke Hu) screened all the titles of the searched studies before the abstract screening. Then, in the second step, another two independent authors checked the abstracts of the studies screened out from the first step (Yining Xu and Kai Xin), while a third independent author (Yanan Gao) checked the full text of the studies screened out from the second step to identify the studies could be finally included in this systematic review.

### 2.6 Data collection process

Two independent authors (Chunxia Ni and Kai Xin) extracted all the original data.

### 2.7 Data items

To compare the pooled effects of different interventions, the details of trials in each included study, namely population, average age, and gender ratio, and intervention protocols with their classification were collected, summarized, and put into an extraction sheet. These outcomes were needed to analyze the heterogeneities within studies and the potential bias of each study as well as the pooled evidence.

To conduct the network meta-analysis, the data of each trial, which contained the sample size N), and mean value (Mean) with its standard deviation (SD) of each outcome in baseline and every recording point, was recorded in another extraction sheet for the data preprocessing. These outcomes were necessary for the identification of inconsistency.

### 2.8 Geometry of the network

The network geometry was made by the Aggregate Data Drug Information System (Version 1.16.8, http://drugis.org/software/addis/index, accessed on 1 October 2022) to display the evidence structure. Each intervention was represented by a node, the direct comparisons between each pair of interventions were represented by the edges, and the arms of each comparison were represented by the number on every edge.

### 2.9 Risk of bias within individual studies

The risk of bias within individual studies was assessed according to the Cochrane Collaboration Risk of Bias Assessment Tool (Armijo-Olivo et al., 2012) in the Cochrane Library Review Manager software (Version 5.3, Wiley, Chichester, United Kingdom) by two independent authors (Yining Xu and Chunxia Ni). An independent arbitrator (Yanan Gao) was invited to arbitrate each disagreement. Cohen’s kappa value was applied to represent the agreement between authors.

If a study had no items with high risk or had less than 3 (contain) items with unclear risk, it was regarded to have a low overall risk. If a study had no item with high risk but had more than 3 items with unclear risk, it was regarded to have a moderate overall risk. If a study had one item with high risk, it was also regarded to have a moderate overall risk. If a study had more than one item with high risk, it was regarded to have a high overall risk.

The reporting bias was also assessed by applying the Cochrane Collaboration Risk of Bias Assessment Tool. If the included study had a pre-registered protocol number and all the outcomes in the protocol were fully matched with those reported in the article, this study was regarded to have a low risk of selective reporting. Meanwhile, if the included study had a pre-registered protocol number but the outcomes reported in the article were not fully matched with those registered in the protocol, this study was regarded as having a high risk of selective reporting. At last, if the included study did not have a pre-registered protocol number, this study was regarded as having an unclear risk of selective reporting (Armijo-Olivo et al., 2012).

### 2.10 Summary measures

The units of f2-isoprostanes, hydrogen peroxide, and MDA in urine, serum, or plasma were different. Therefore, in this systematic review, the effect was presented in the form of standardized mean differences and their standard error (SMD ± SE). According to the criteria provided by Cohen, when SMD was larger than 0.8, the effect size was large, when the SMD was from 0.5 to 0.8, the effect size was moderate when the SMD was from 0.2 to 0.5, the effect size was small, and when the SMD was less than 0.2, the effect size was very small (Gogtay and Thatte, 2017).

Under the consistency model, the results were shown in a rank probability plot, in which the sum of all rank probabilities was 1. In a rank probability plot, a lower rank number meant the induction of lower oxidative stress. Therefore, in the probability rank of interventions, the intervention in Rank N would induce a higher cellular lipid oxidative stress. A league table was provided after the model of data analysis had been determined and reported the results that showed the standard mean difference in the column-defining items compared with the row-defining items.

Under the inconsistency model, the results were shown only by a league table (Catala-Lopez et al., 2014).

### 2.11 Planned methods of analysis

Two independent authors (Yanan Gao and Chunxia Ni) conducted the data preprocessing and analysis. Microsoft Office Excel (Version 16.0, Microsoft Corporation, Redmond, WA, United States) was applied to transfer original outcomes to SMDs and their SE. The Aggregate Data Drug Information System (Version 1.16.8, http://drugis.org/software/addis/index, accessed on 1 October 2022) was used to make the network meta-analysis.

### 2.12 Assessment of inconsistency

If there are closed loops in the intervention structure, the inconsistency of the evidence must be assessed. The node-splitting analysis is an alternative method to assess inconsistency in network meta-analysis, which assesses whether direct and indirect evidence on a specific node (the split node) agree when the results are easier to interpret and require a separate model to be run for each node to be split (Rouse et al., 2017).

The consistency model was applied if there were neither closed loops nor split nodes in the intervention structure, the respective Bayesian *p*-value in every node-splitting analysis was larger than 0.05, or the random-effects standard deviations in the two models were identical. Otherwise, the inconsistency model should be applied (Catala-Lopez et al., 2014).

### 2.13 Risk of bias across studies

Two independent authors (Yining Xu and Keke Hu) applied the Cochrane Collaboration Risk of Bias Assessment Tool (Armijo-Olivo et al., 2012) in the Cochrane Library Review Manager software (Version 5.3, Wiley, Chichester, United Kingdom) to assess the risk of bias across studies.

### 2.14 Additional analyses

The confidence evaluation and the reporting bias assessment were conducted by applying The Confidence in Network Meta-Analysis (CINeMA https://cinema.ispm.unibe.ch, assessed on 1 October 2022). The confidence should be downgraded by one level when the item “within-study bias” was a “Major concern” or the other items were “Some concern”. The confidence would be downgraded by two-level when other items were “Major concern” (Nikolakopoulou et al., 2020; Papakonstantinou et al., 2020).

The summarizing risk of bias assessments was conducted according to the “Average Risk of Bias” setting in CINeMA. The weighted average score for each relative effect was estimated according to the percentage contribution of studies at each bias level. Studies of a direct comparison, which had low (arbitrarily assigned a score of 1), moderate (score 2), and high (score 3) risk of bias, had 40%, 25%, and 35% percentage of contribution, and the total risk of bias score was 0.40 × 1 + 0.25 × 2 + 0.35 × 3 = 1.95, which rounded to 2 and would be regarded as “Some concerns” (Nikolakopoulou et al., 2020; Papakonstantinou et al., 2020).

## 3 Results

### 3.1 Study selection

**FIGURE 1:**
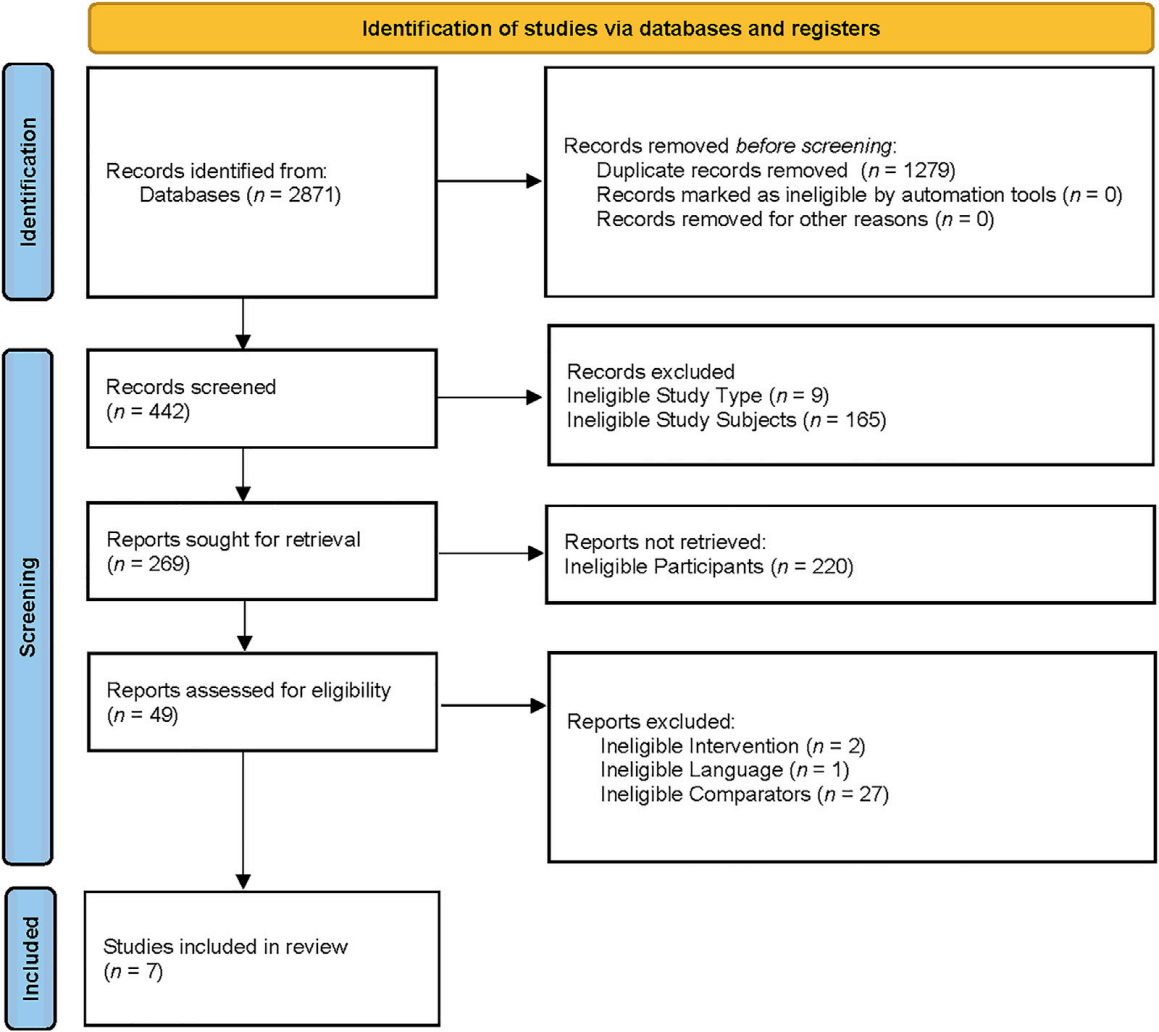
The PRISMA 2020 flow diagram for new systematic reviews which included searches of databases and registers only.

The search yielded 2,871 titles and abstracts for screening. After removing 1,279 duplicated studies, 442 studies were included in the records screening process. In the records screening process, 9 studies that were not RCTs, 165 studies of animal trials, and 220 studies with wrong participants were excluded. The remaining 49 studies were included for full-text screening. Among the 49 studies, 2 studies were excluded due to their ineligible interventions, 1 study was excluded because of its ineligible language, and 27 studies were excluded because of their ineligible comparators. Eventually, 7 studies were included in the systematic review (Alghadir et al., 2016; Campbell et al., 2010; da Silva et al., 2021; Dani et al., 2020; Fatouros et al., 2004; Osali, 2020; Vincent et al., 2002). The flow diagram was presented in Figure 1.

### 3.2 Study characteristics

**FIGURE 2:**
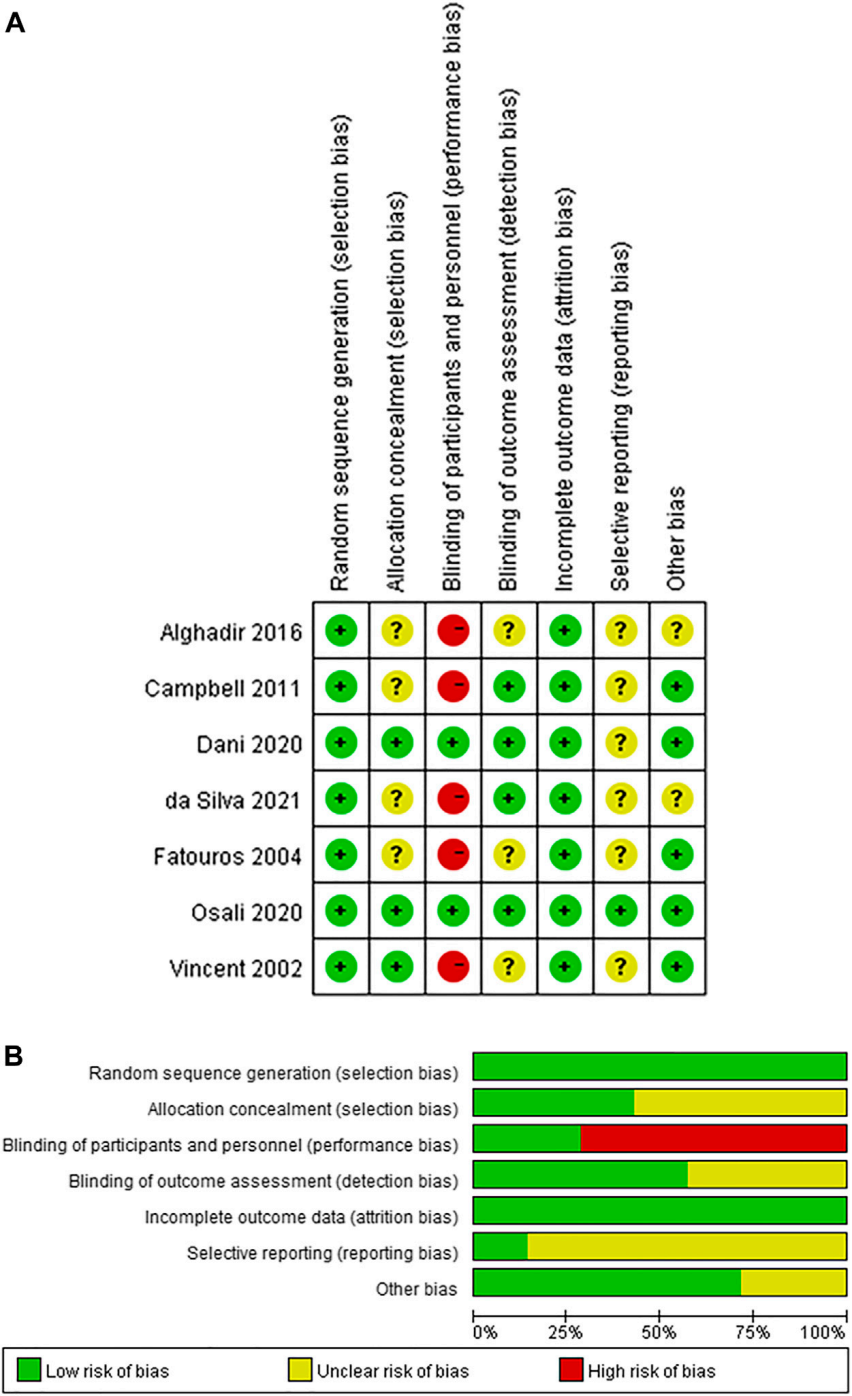
The result of the risk of bias assessment **(A)** Risk of bias summary **(B)** Risk of bias graph.

Two studies recruited unhealthy elders as participants, while 5 studies recruited healthy or healthy elders as participants. 1 study used F2-isoprostane as the outcome measure, 1 study used LIPOX as the outcome measure, and others used MDA as the outcome measure. Only 2 studies give their participants antioxidant supplementation during their trials. Detailed information on all included studies is provided in Table 1, and the results of the quality assessment was provided in Figure 2.

**TABLE 1:** The detailed information in included trials.

Study	Participants			Intervention				Outcome	
	Description	Age	Gender (F/M)	Description	Detail	Classification	Duration	Parameters (Units)	Main results
Campbell 2020 (Campbell et al., 2010)	Overweight or obese, postmenopausal, sedentary women	60.65	173/0	Moderate-intensity aerobic exercise	60%–75% HRmax, ≥45min/day, 5 days/week	AE	12 months	Urinary F2-isoprostane (mg/mg creatinine)	AE group: 6.2%
									NE group: + 3.3%
				Stretching	1/week, 45 min/per, stretching and relaxation, asked to not otherwise change exercise habits	NE			Without statistical significance between groups (*p* = 0.26)
Alghadir 2016 (Alghadir et al., 2016)	Healthy Older Adults	69.7	35/100	Moderate-intensity aerobic exercise	60%–70% HRmax, 45–60min, 3 days/week	AE	24 weeks	Serum MDA (μmol/L)	AE group: 15.5 ± 6.7 to 5.1 ± 1.8, (*p* < 0.01)
				Non-exercise	Without any exercise	NE			NE group: from 4.7 ± 3.5 to 2.7 ± 1.7, (*p* < 0.05)
Fatouros 2004 (Fatouros et al., 2004)	Older Men	72.25	0/19	Aerobic Endurance Training	3 training sessions/week, walking/jogging at 50%–80% of HRmax	AE	16 weeks	Plasma MDA (μM)	AE group: 1.02 ± 0.11 to 0.85 ± 0.12, (*p* < 0.05)
				Non-exercise	Without any exercise	NE			NE group: 1.01 ± 0.12 to 1.04 ± 0.10, (*p* > 0.05)
da Silva 2021 (da Silva et al., 2021)	Older Hemodialysis Patients with chronic kidney diseases	67	71/157	Resistance Training	3 sets of 8–12 repetitions with RPE from 5 to 8, 12 exercises, over 40 min, 3 weekly	LIRT	6 months	Serum MDA (mM)	LIRT group: 14.1 ± 2.3 to 11.0 ± 2.9, (*p* < 0.05)
				Non-exercise	Not revive any exercise intervention	NE			NE group: 13.2 ± 2.4 to 13.6 ± 2.6, (*p* > 0.05)
									With statistical significance between groups (*p* < 0.05)
Dani 2020 (Dani et al., 2020)	Healthy elderly women	69.75	29/29	Grape Juice	400 ml grape juice/day	S	1 month	Plasma TBARS (nmol/mg)	Grape juice group: 3.23 ± 2.03 to 4.75 ± 1.32, (*p* > 0.05)
				Placebo and Exercise	400 ml placebo/day + 2/week, 60 min concurrent physical training with resistance and aerobic training	Placebo + AE + LIRT			Placebo and exercise group: 2.38 ± 0.82 to 2.57 ± 0.60 (*p* > 0.05)
				Grape Juice and Exercise	400 mL grape juice/day + 2/week, 60 min concurrent physical training with resistance and aerobic training	S + AE + LIRT			Grape juice and exercise group: 3.11 ± 1.07 to 3.01 ± 0.67, (*p* > 0.05)
Osali 2020 (Osali, 2020)	Elderly females with metabolic syndrome	62.3	44/44	Aerobic Exercise + Nano-curcumin supplementation	Walked or ran at 65%–75% heart rate reserve (HRR) on a treadmill for 3 × 12—17 min + 80 mg/day supplementation	AE + S	6 weeks	Plasma MDA (nmol/dL)	AE + S group: 2.68 ± 0.71 to 1.47 ± 0.62, (*p* < 0.05)
				Aerobic Exercise	Walked or ran at 65%–75% heart rate reserve (HRR) on a treadmill for 3 × 12—17min	AE			AE group: 2.74 ± 0.78 to 1.42 ± 0.68, (*p* < 0.05)
				Nano-curcumin supplementation	80 mg/day supplementation	S			S group: 2.59 ± 0.82 to 1.32 ± 0.78, (*p* < 0.05)
				Placebo	80 mg/day maltodextrin placebo	Placebo			Placebo group: 2.79 ± 0.61 to 2.81 ± 0.8, (*p* > 0.05)
Vincent 2002 (Vincent et al., 2002)	Apparently healthy adults aged between 60 and 83 years	68.12	Unknown	Non-exercise	Without any exercise	NE	6 weeks	LIPOX (nmol/mL)	NE group: 0.24 ± 0.05 to 0.23 ± 0.05, (*p* > 0.05)
				Low-intensity Resistance Exercise	50% one-repetition maximum (1 R M), 13 repetitions/exercise	LIRT			LIRT group: 0.23 ± 0.05 to 0.24 ± 0.05, (*p* > 0.05)
				High-intensity Resistance Exercise	80% 1 R M, 8 repetitions/exercise	HIRT			HIRT group: 0.25 ± 0.06 to 0.23 ± 0.05, (*p* > 0.05)

AE: aerobic exercise; NE: non-exercise; S: antioxidant supplementation; LIRT: low-intensity resistance training; HIRT: high-intensity resistance training.

### 3.3 Results of syntheses

#### 3.3.1 Evidence structure

**FIGURE 3:**
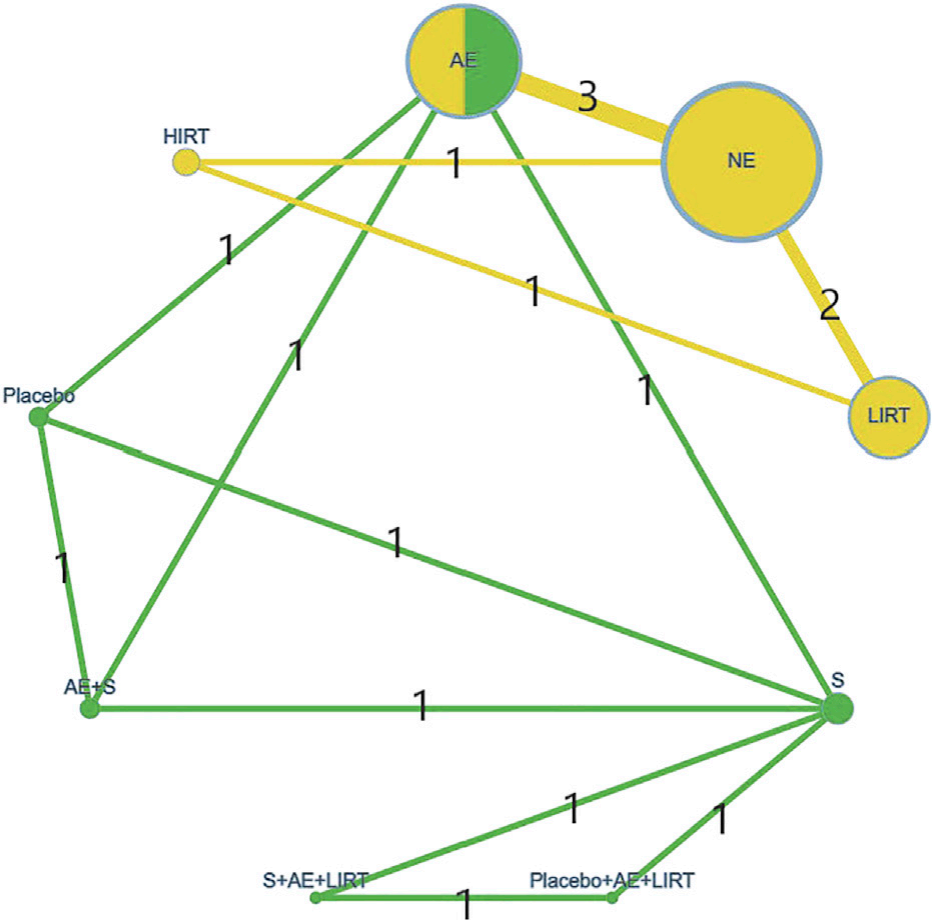
The network geometry of the interventions.

In the evidence structure, which was represented by a network geometry (Figure 3), the risk of bias distribution of each intervention was the color in every node (Red = high risk of bias, Yellow = unclear risk of bias, Green = low risk of bias), the sample size of each intervention was the size of the node, and the number of arms in each direct comparison was the width of the lines that connected two interventions and was also equal to the numbers on them.

#### 3.3.2 Network meta-analysis

The results of consistency tests and node splitting analysis were presented in Table 2. According to Table 2, the random-effects standard deviation of consistency was 1.47 (95%CI: 0.73 to 2.20), while that of the inconsistency model was 1.39 (95%CI: 0.74 to 2.20). Moreover, the result of the node splitting analysis showed that the direct and indirect effect of the comparison between intervention AE and S had no statistically significant difference (*p* = 1.00). Since the random-effects standard deviations calculated under consistency and inconsistency models were identified and then the *p*-value of node splitting analysis was larger than 0.05, the consistency model was appropriate to conduct the network meta-analysis.

**TABLE 2:** The results of consistency tests and node splitting analysis.

Model test	Model	Random-effects standard deviation	
	Consistency	1.42 (0.73, 2.20)	
	Inconsistency	1.39 (0.74, 2.20)	
Node splitting	Intervention	Overall Effect	*p*-value
	AE, S	−0.22 (−3.34, 2.97)	1.00

AE: aerobic exercise; S: antioxidant supplementation.

Table 3 was the league table that showed the weighted standard mean difference calculated by the effect size of column-defining interventions minus that of the row-defining interventions. According to Table 3, the intervention categories, namely LIRT + AE + Placebo and LIRT + AE + S, both reduced cellular lipid peroxidation compared with all other intervention categories, since the values in the row cells, which represented the effects of the interventions in the row headers minus those of the interventions in the column headers for that cells, were all less than 0.

**TABLE 3:** The league tables of the network geometries.

AE	0.72	0.35	0.00	−0.27	−0.13)	−1.06	−1.78	−0.22
	AE + LIRT + Placebo	−0.34	−0.69	−0.95	−0.85	−1.78	−2.49	−0.94
		AE + LIRT + S	−0.36	−0.61	−0.47	−1.43	−2.15	−0.60
			AE + S	−0.26	−0.12	−1.08	−1.79	−0.23
				HIRT	0.11	−0.82	−1.55	0.04
					LIRT	−0.95	−1.66	−0.09
						NE	−0.70	0.85
							Placebo	1.57
								S

AE: aerobic exercise; NE: non-exercise; S: antioxidant supplementation; LIRT: low-intensity resistance training; HIRT: high-intensity resistance training.

**FIGURE 4:**
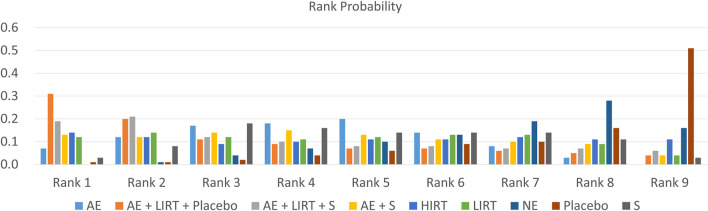
Ranking of measures and probabilities.

Figure 4 and Table 4 provided the probability rank of every intervention, in which a lower rank number meant a dampening of cellular lipid peroxidation, and a higher rank number indicated an enhancement of cellular peroxidation. According to Table 4, for elder adults, a combination program of aerobic exercise, low-intensity resistance training, and a placebo intake and a combination program of aerobic exercise, low-intensity resistance training, and an antioxidant supplementation had the most and sub-most potential to dampen cellular lipid peroxidation (AE + LIRT + Placebo: 0.31 in Rank 1 and 0.2 in Rank 2; AE + LIRT + S: 0.19 in Rank 1 and 0.20 in Rank 2). Besides, a placebo intake control intervention and a blank intervention without exercise had the most and sub-most potential to induce an enhancement of cellular lipid peroxidation (Placebo: 0.51 in Rank 9 and 0.16 in Rank 8; NE: 0.16 in Rank 9 and 0.28 in Rank 8).

**TABLE 4:** Ranking of measures and probabilities^a^.

Intervention	Rank 1	Rank 2	Rank 3	Rank 4	Rank 5	Rank 6	Rank 7	Rank 8	Rank 9
AE	0.07	0.12	0.17	0.18	0.20	0.14	0.08	0.03	0.00
AE + LIRT + Placebo	0.31	0.20	0.11	0.09	0.07	0.07	0.06	0.05	0.04
AE + LIRT + S	0.19	0.21	0.12	0.10	0.08	0.08	0.07	0.07	0.06
AE + S	0.13	0.12	0.14	0.15	0.13	0.11	0.10	0.09	0.04
HIRT	0.14	0.12	0.09	0.10	0.11	0.11	0.12	0.11	0.11
LIRT	0.12	0.14	0.12	0.11	0.12	0.13	0.13	0.09	0.04
NE	0.00	0.01	0.04	0.07	0.10	0.13	0.19	0.28	0.16
Placebo	0.01	0.01	0.02	0.04	0.06	0.09	0.10	0.16	0.51
S	0.03	0.08	0.18	0.16	0.14	0.14	0.14	0.11	0.03

AE: aerobic exercise; NE: non-exercise; S: antioxidant supplementation; LIRT: low-intensity resistance training; HIRT: high-intensity resistance training.

^a^ Rank 1 and Rank 9 mean the most and least positive effect on dampening cellular lipid peroxidation.

### 3.4 Reporting bias

The results of the reporting bias assessment were provided in the risk of bias assessment results in Figure 2. According to Figure 2, all included studies had an unclear risk of selecting reporting since all the trials in these included studies were not registered.

### 3.5 Certainty of evidence

Table 5 provided the results of the confidence assessment made by CINeMA. In Table 5, the “MC” indicates the issue needs major concern, the “SC” indicates the issue needs some concern, and the “NC” indicates the issue needs no concern. According to Table 5, there were no high confidence ratings in all the direct and indirect comparisons, the comparisons between AE and Placebo, AE + S and Placebo, LIRT and NE, and Placebo and S in the direct evidence structure had moderate confidence ratings, whereas, the comparisons between AE and LIRT, A + S and LRT, LIRT and Placebo, LIRT and S, NE and Placebo, NE and Placebo + AE + LIRT, Placebo and S + AE + LIRT in the indirect evidence structure had moderate confidence.

**TABLE 5:** Results of the confidence rating.

Structure	Comparison	Number of studies	Within-study bias	Reporting bias	Indirectness	Imprecision	Heterogeneity	Incoherence	Confidence rating
Direct	AE:AE + S	1	NC	SC	NC	MC	NC	NC	Very low
	AE:NE	3	SC	SC	NC	NC	MC	NC	Very low
	AE:Placebo	1	NC	SC	NC	NC	NC	NC	Moderate
	AE:S	1	NC	SC	NC	MC	NC	NC	Very low
	AE + S:Placebo	1	NC	SC	NC	NC	NC	NC	Moderate
	AE + S:S	1	NC	SC	NC	MC	NC	NC	Very low
	LIRT:NE	2	SC	SC	NC	NC	NC	NC	Moderate
	Placebo:S	1	NC	SC	NC	NC	NC	NC	Moderate
	Placebo + AE + LIRT:S	1	NC	SC	NC	NC	MC	NC	Very low
	Placebo + AE + LIRT:S + AE + LIRT	1	NC	SC	NC	MC	NC	NC	Very low
	S:S + AE + LIRT	1	NC	SC	NC	NC	MC	NC	Very low
Indirect	AE:LIRT	0	SC	SC	NC	NC	NC	NC	Moderate
	AE:Placebo + AE + LIRT	0	NC	SC	NC	NC	MC	NC	Very low
	AE:S + AE + LIRT	0	NC	SC	NC	MC	NC	NC	Very low
	AE + S:LIRT	0	SC	SC	NC	NC	NC	NC	Moderate
	AE + S:NE	0	NC	SC	NC	MC	NC	NC	Very low
	AE + S:Placebo + AE + LIRT	0	NC	SC	NC	NC	MC	NC	Very low
	AE + S:S + AE + LIRT	0	NC	SC	NC	NC	NC	NC	Very low
	LIRT:Placebo	0	SC	SC	NC	NC	MC	NC	Moderate
	LIRT:Placebo + AE + LIRT	0	NC	SC	NC	MC	NC	NC	Very low
	LIRT:S	0	SC	SC	NC	NC	NC	NC	Moderate
	LIRT:S + AE + LIRT	0	NC	SC	NC	SC	SC	NC	Very low
	NE:Placebo	0	NC	SC	NC	NC	NC	NC	Moderate
	NE:Placebo + AE + LIRT	0	NC	SC	NC	NC	NC	NC	Moderate
	NE:S	0	NC	SC	NC	SC	SC	NC	Very low
	NE:S + AE + LIRT	0	NC	SC	NC	NC	SC	NC	Low
	Placebo:Placebo + AE + LIRT	0	NC	SC	NC	NC	NC	NC	Moderate
	Placebo:S + AE + LIRT	0	NC	SC	NC	NC	NC	NC	Moderate

AE: aerobic exercise; NE: non-exersice; S: antioxidant supplementation; LIRT: low-intensity resistance training; HIRT: high-intensity resistance training; NC: no concern; SC: some concern; MC: major concern.

## 4 Discussion

The main findings of this systematic review and network meta-analysis can be concluded as follows. Firstly, a long-term combined training program that includes aerobic and resistance exercise and lasts more than 4 weeks, could induce a dampening of the cellular lipid peroxidation in the bodies of elder individuals, regardless of their intake of antioxidant supplementation. Second, a daily life routine without exercise might have an enhancing effect on cellular lipid peroxidation in the bodies of elderly individuals, since a placebo intake protocol intervention without exercise and a blank intervention without exercise had the most and sub-most potential to induce an enhancement of cellular lipid peroxidation, respectively.

Many previous studies have demonstrated the positive effect induced by resistance training and aerobic exercise on intercellular antioxidation, which correspondent with one of the main findings of this systematic review. Some of these studies were about animal trials. For example, a study published in 2016 by da Palma’s team, whose subjects were rats, claimed that both resistance and aerobic exercise could induce an improvement in cardiac oxidative stress (da Palma et al., 2016), and another rat trial conducted by Quinteiro’s team in 2015 demonstrated that aerobic and resistance exercise training could bring a reduction in oxidative stress in an experimental model of diabetes and menopause (Quinteiro et al., 2015). Regarding elderly human beings, in 2012, Cardoso’s team identified that, when compared with sedentary middle-aged women, trained middle-aged women show improved antioxidant capacity and lower oxidative damage, demonstrating the benefits of chronic regular physical activity (Cardoso et al., 2012), and a pilot study conducted by Done’s team in 2016, whose subjects were older and middle-aged individuals from 50 to 63 years old, demonstrated that regular aerobic and resistance exercise can increase their resistance to oxidative stress (Done and Traustadottir, 2016).

Additionally, these findings indicated that regular resistance exercise under a low intensity might bring more advantages in the antioxidation of cellular lipids. This finding also correspondent with some previous studies. A study published in 2016 found that, for hypertensive elderly women, a strength training program could reduce oxidative stress and correlate moderately with cardiovascular benefits (Dantas et al., 2016). And a study published in 2022 by Rodziewicz-Flis’ team demonstrated that a 12-week training contained low-intensity resistance exercise improved physical performance and antioxidant protection in elderly women (Rodziewicz-Flis et al., 2022).

Besides, several previous studies have identified that cellular lipid peroxidation would be gradually more intense with the body’s senility. For example, a cross-sectional study published in 2011 found that athletes had higher endogenous antioxidant protection than sedentary individuals and the potential mechanism might be that chronic exercise could protect against exercise-induced oxidative stress by upregulating endogenous antioxidant defense systems (El Abed et al., 2011). At the same time, a study published in 2020, whose participants were obese men, identified that exercise can change the redox state and enhance the brain-derived neurotrophic factor production by lipopolysaccharide-stimulated oxidative stress markers of peripheral blood mononuclear cells in obese individuals (Elsner et al., 2020). These physiological and biochemical mechanisms might partially explain the findings of this systematic review that if an elderly individual had daily life without any exercise, the cellular lipid peroxidation in his or her body would be enhanced more.

However, the results of the network meta-analysis in this systematic review seem not to support the positive effect of anti-oxidant supplementation for cellular lipid peroxidation in elderly individuals, since according to the rank probabilities of interventions, the interventions containing anti-oxidant supplementation did not have any obvious advantage in becoming the best intervention to dampen cellular lipid peroxidation when being compared with other protocols without anti-oxidant supplementation, and might even have disadvantages when being compared with protocols without scheme without anti-oxidant supplementation when elderly individuals have a regular aerobic and resistance exercise (AE + LIRT + Placebo: 0.31 in Rank 1 and 0.2 in Rank 2; AE + LIRT + S: 0.19 in Rank 1 and 0.20 in Rank 2). This finding correspondent with what has been mentioned by Ristow’s study, which is that the enhancement of antioxidation will be affected by anti-oxidation supplementation and then the potential benefit of exercise will be decreased (Ristow et al., 2009). Therefore, this result indicates that the importance of exercise for preventing oxidative stress is similar in the elder and physically active individuals and further demonstrates the importance of keeping a high level of daily activity for the elderly, especially those physical activities that contain both aerobic and resistance exercise.

Nevertheless, this viewpoint that the intake of anti-oxidant supplementation has a negative effect on individuals with exercise still needs to be considered dialectically, and the reason might come from the following perspectives. First, at present, most evidence that supports the application of antioxidative supplements is still at a low level. Although several controlled trials with human or animal subjects have demonstrated the positive effects of antioxidant supplementation for oxidative stress (Kooshki et al., 2020; N'Guessan B et al., 2022; Onaolapo et al., 2021; Sharma et al., 2020), there is still no systematic review with meta-analysis to identify which antioxidant supplementation protocol would be the best choice for individuals in different ages. Second, generally, the antioxidant supplements given to subjects in studies are often composite components, making it difficult to identify the specific contribution of each component to the antioxidant physiological mechanism and whether they would interact with each other. At last, the publication bias of the studies on the antioxidant supplements cannot be ignored since the potential conflict of interest in the relevant studies and the absence of grey literature of trials with small effect sizes cannot be excluded.

There are some limitations to this systematic review and network meta-analysis. First, none of the included studies registered for clinical trials, making the reporting bias unclear, inducing a downgrading of the confidence rating for the results. Second, only 2 included studies reported the change in cellular lipid peroxidation indicators after an antioxidant supplementation. The antioxidant supplementation protocols of these 2 studies were different. Therefore, it was possible that the dampening effect of antioxidant supplementation on cellular lipid peroxidation did not show advantages in this systematic review due to the heterogeneity within supplementation protocols. Third, although there was no significant inconsistency found in the evidence structure, the intervention durations and exercise protocols of included studies were different that the heterogeneities within included studies could not be ignored. Lastly, 3 studies recruited elderly participants with obesity, metabolic syndrome, and chronic kidney disease, making it possible that there was a potential heterogeneity induced by the different participants of studies.

## 5 Conclusion

To sum up, a long-term combined training program that includes aerobic and resistance exercise could induce a dampening of the cellular lipid peroxidation in the bodies of elder individuals, regardless of their intake of antioxidant supplementation, and a daily life routine without exercise might have an enhancing effect on cellular lipid peroxidation in the bodies of elderly individuals, being harmful to their health, respectively.

## Data Availability

The original contributions presented in the study are included in the article/Supplementary Material, further inquiries can be directed to the corresponding author.
